# High prevalence of *Giardia* spp. in cats without diarrhea and agreement between diagnostic techniques in the municipality of Londrina, Paraná, Brazil

**DOI:** 10.1007/s11259-026-11409-8

**Published:** 2026-07-20

**Authors:** Luiz Roberto Santos Aoki, Fernando de Souza Rodrigues, Giovanna Cristina Justino Santos Aoki, Lethicia Mendes Nino, Mateus Siqueira Pyles, Michel dos Santos Pinto, Michelle Santos Sabioni, João Alfredo Biagi Camargo Neto, Alex Akira Nakamura, Katia Denise Saraiva Bresciani, Gustavo Felippelli, Marcos Franke Pinto

**Affiliations:** 1 University Center of Technology of Curitiba, UNIFATEC, Curitiba, Paraná Brazil; 2 Londrina State University, UEL, Londrina, Paraná Brazil; 3 School of Veterinary Medicine, São Paulo State University, Unesp, Araçatuba, São Paulo Brazil

**Keywords:** Felines, Hexamitidae, Protozoa, Zoonoses

## Abstract

In this study, we conducted an epidemiological investigation and analyzed the agreement between the Faust technique and the immunochromatographic assay in the diagnosis of *Giardia* spp. in 272 cats in the municipality of Londrina, Paraná state, Brazil. We identified an overall prevalence of 74.63% (203/272 [95% CI: 69.14–79.44%]) of *Giardia* spp. in cats, being significantly higher in adult and non-domiciled animals, which presented, respectively, 5.3 and 8.6 times more chances of infection when compared to kittens and domiciled animals. However, all the animals investigated that tested positive did not present with diarrhea. Regarding diagnostic methods, using coproparasitological examination, *Giardia* spp. cysts were observed in the feces of 193 cats, and through the immunochromatographic assay, coproantigens of this protozoan were detected in 203 samples, demonstrating almost perfect agreement (*Kappa* Cohen: 0.907). Thus, according to the authors’ knowledge, this is the highest prevalence of *Giardia* spp. ever identified in cats in Brazil, and with regard to diagnostic methods, we observed a high concordance between the coproparasitological examination and the immunochromatographic assay in the detection of the aforementioned protozoan.

**Supplementary Information:**

The online version contains supplementary material available at https://doi.org/10.1007/s11259-026-11409-8.

## Introduction

Since 1979, the World Health Organization (WHO) has recognized giardiasis as a zoonotic disease (Levine 1980), and it is considered a Neglected Tropical Disease (Savioli et al. 2006). It is worth noting that the aforementioned protozoan is considered the most common parasite in small animal clinical practice, particularly due to the difficulty in its eradication, especially in overpopulated environments, as it causes diarrheal diseases in animals and humans (Ryan et al. 2021). Annually, approximately 490,000 children die from diarrheal diseases worldwide, with *Giardia* spp. being one of the main parasites associated with these fatal outcomes (WHO 2024).

Giardiasis is a parasitic disease caused by species of the protozoan *Giardia* spp., with mammals, including humans, being primarily affected by *Giardia duodenalis* (syn. *Giardia lamblia* and *Giardia intestinalis*). This species has a wide genetic variability, divided into eight genotypes called assemblages ranging from A-H, of which A and B are considered zoonotic because they affect a wide range of animals and humans, and the others are typical of various animal species, with felines generally affected by assemblage F (Ryan et al. 2021). However, in Ethiopia (Gelanew et al. 2007), Slovakia (Pipiková et al. 2020) and Brazil (Santos Silva et al. 2022), humans have already been identified with the F assemblage, and the identification of these atypical genotypes suggests a new route in the zoonotic transmission of *Giardia duodenalis* (Fantinatti et al. 2016).

The diagnosis of *Giardia duodenalis* infection can be performed using different approaches, including microscopic detection of cysts and trophozoites and immunological assays targeting parasite antigens. Microscopic techniques are inexpensive and widely available but may exhibit reduced sensitivity due to intermittent cyst shedding. In contrast, immunological assays generally provide greater sensitivity and do not depend on the observer’s expertise, although differences in diagnostic performance among available tests have been reported. Therefore, the simultaneous application of both methods and the evaluation of their agreement may contribute to a more accurate assessment of *Giardia duodenalis* occurrence in epidemiological studies (Uchôa et al. 2017; de Mendonça Uchôa et al. 2018). Thus, the aim of this study was to investigate the prevalence of *Giardia duodenalis* in felines in the municipality of Londrina, Paraná, Brazil, and to assess the agreement between the Faust technique and the immunochromatographic assay in the diagnosis of this protozoan.

## Materials and methods

### Study area and population

**Fig. 1:**
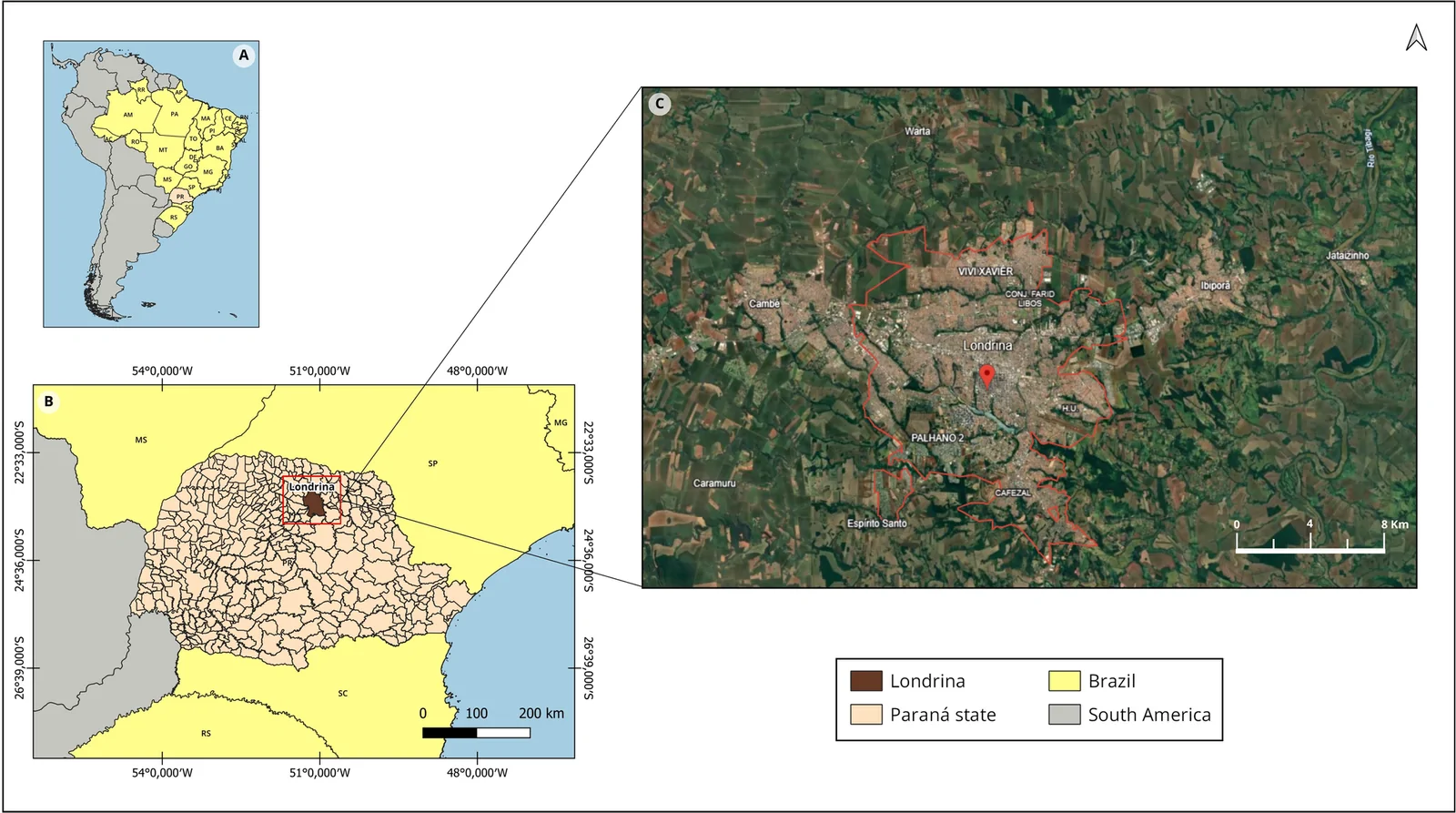
Study area. Map of Brazil (**A**) and the state of Paraná (**B**), showing the municipality of Londrina (**C**). Map created with QGIS 3.28.9 software, using freely accessible shapefiles from the Brazilian institute of geography and statistics (IBGE) from 2023. Image **C** was generated using google earth

In this research, the sample size was calculated according to Cochran’s formula, n = Z^2⋅p⋅(1-p)/e^2, considering an infinite population (Cochran 1977), resulting in a minimum sample size of approximately 246 felines. The confidence level was 95% (1.96 [Z]), with an expected prevalence of 20% (p) and a margin of error of 5% (e). Thus, a total of 272 felines from the municipality of Londrina, Paraná state, Brazil (Fig. 1), were investigated. These animals were characterized according to age range and rearing system (Table 1). However, information regarding the breed and sex of the animals was not collected.

The non-domesticated animals came from four Non-Governmental Organizations (NGOs).

**Table 1:** Characterization of felines investigated for *Giardia* spp., according to age group and rearing in the municipality of Londrina, state of Paraná, Brazil

Cats	Animals		Total (%)
	NGOs (%)	Domiciled (%)	
Kittens (≤ 1 year)	8 (40)	12 (60)	20 (7.4)
Adults (> 1 year)	196 (77.8)	56 (22.2)	252 (92.6)
**Total (%)**	204 (75)	68 (25)	272 (100)

### Sample collection

A single fecal sample from each feline was investigated for the presence of *Giardia* spp. Samples were collected after spontaneous defecation by the animals’ respective owners in the case of domiciled animals, and by the researchers of this study in the case of NGOs animals. The feces were placed in collection containers, identified, and sent for processing.

It is important to note that animals presenting liquid or pasty feces, consistent with diarrhea, were classified as having clinical signs of giardiasis, whereas those with solid feces, without diarrheic characteristics, were classified as not presenting clinical signs.

### Diagnosis of *Giardia* spp.

#### Flotation technique

The parasitological detection of *Giardia* spp. cysts was performed using the Faust technique (Faust et al. 1938).

#### Immunochromatography test

The diagnosis of *Giardia* spp. coproantigens was performed using the *Giardia* Ag VET FAST test (Bioclin Vet, MG, Brazil) according to the manufacturer’s protocol. According to the manufacturer’s data, the immunological test used in our study has a sensitivity of 97.83% (95% CI: 88.47%–99.94%) and a specificity of 96.83% (95% CI: 89.00%–99.61%).

### Statistical analysis

The prevalence with a 95% Confidence Interval (CI) was calculated using Wilson’s method, and the analysis of the results consisted of descriptive and inferential statistics, using Fisher’s Exact Test, correlating the positivity and negativity of the animals with the main variables, such as age group (puppies and adults) and rearing system (domiciled and NGOs), with significance considered when *P* < 0.05.

To verify the level of agreement between the diagnostic techniques used (Faust technique and immunochromatographic assay), Cohen’s *Kappa* coefficient was calculated. The coefficient classification was performed according to the following intervals: from 0 to 0.19 (poor agreement); 0.20 to 0.39 (fair agreement); 0.40 to 0.59 (moderate agreement); 0.60 to 0.79 (substantial agreement), and 0.80 to 1.00 (almost perfect agreement) (Landis and Koch 1977).

## Results

In this study, of the 272 felines investigated, 203 were positive for *Giardia* spp., representing a prevalence of 74.63% (95% CI [69.14–79.44%]). However, none of the animals presented with diarrhea. Among the positive animals, *Giardia* spp. was detected in 193 cats through parasitological examination and in 203 by immunochromatographic assay, with almost perfect agreement according to Cohen’s *Kappa* coefficient (0.907) additional data are given in Online Resource 1. The prevalence of *Giardia* spp., according to the variables and diagnostic tests, can be observed in Online Resource 2.

Regarding the statistical analysis, in this study, we observed a significantly higher prevalence of *Giardia* spp. in adult felines, with these animals having 4.1 times more chances of infection by the aforementioned protozoan when compared to kittens. Correlating the rearing system, we observed that NGOs animals presented a significantly higher prevalence, with 8.6 times more chances of infection when compared to domiciled felines (Table 2).

**Table 2:** Correlation of *Giardia* spp. prevalence in relation to age group and feline rearing system in the municipality of Londrina, Paraná state, Brazil

Variables		N° of investigated animals	N° of positive animais	Prevalence (%)	Confidence interval (95%)	Fisher’s exact test (P value)	Odds ratio
Age group	Kittens	20	9	45	25.82–65.79	*P* = 0.0054*	0.2446
	Adults	252	194	76.98	71.41–81.75		
Rearing system	Domiciled	68	28	41.18	30.26–53.04	*P* < 0.0001*	0.1160
	NGOs	204	175	85.78	80.33–89.92		

Nº - Number

* - Statistically significant

## Discussion

In this research, the prevalence of *Giardia* spp. in cats without diarrhea was 74.63%, being significantly higher in adult and NGOs. According to Cohen’s *Kappa* coefficient, the Faust technique and the immunochromatographic assay showed almost perfect agreement. In Brazil, using parasitological techniques, prevalences lower than that found in this study were reported in the municipalities of São Paulo, with 5.2% (26/502) of positive felines (Gennari et al. 2016), in Londrina with 6.31% (13/206) (Torrico et al. 2020), in Santos with 13.33% (2/15) (Lima et al. 2021) and Jataí with 3.64% (2/55) (Souza et al. 2023). Furthermore, through Polymerase Chain Reaction, in Londrina 17.96% (37/206) of feline samples were positive (Torrico et al. 2020), with the results obtained in this study being the highest prevalence detected in felines in the country to date.

In this study, none of the positive felines presented with diarrhea; however, the animals released cysts in their feces and acted as a reservoir for *Giardia* spp. It is worth highlighting that the B assemblage of *G. duodenalis* is the most common genotype in human infections (Ryan et al. 2021); however, the F assemblage, a genotype commonly diagnosed in felines, has also been identified in humans in Ethiopia (Gelanew et al. 2007), Slovakia (Pipiková et al. 2020), and Brazil (Santos Silva et al. 2022), thus emphasizing the capacity of these animals as a source of infection for zoonotic genotypes.

In our research, we observed a significantly higher prevalence of *Giardia* spp. in non-domiciled felines, and this result increases the importance of giardiasis in the context of Public Health, since stray animals are frequently adopted by families with children, which can potentiate zoonotic transmission (Fantinatti et al. 2018).

In this research, we identified a significantly higher prevalence of *Giardia* spp. in adult felines. The higher prevalence of *Giardia spp.* observed in non-domiciled cats may be explained by increased exposure to contaminated environments. Stray animals are more frequently subjected to inadequate sanitary conditions, high population density, and constant contact with contaminated water, food sources, and feces from other animals, which facilitates the ingestion of infective cysts (Bouzid et al. 2015; Ryan et al. 2021).

Regarding the higher prevalence in adult felines, although younger animals are often described as more susceptible to infection (Bouzid et al. 2015), this finding may be associated with cumulative exposure over time, increasing the likelihood of infection in older animals. Furthermore, cats may shed cysts intermittently without showing clinical signs, contributing to the maintenance of the parasite in the environment (Uchôa et al. 2017; Ryan et al. 2021).

The detection of the aforementioned protozoan in felines in the municipalities of São Paulo (Gennari et al. 2016) and Santos (Lima et al. 2021) did not show statistical significance in relation to age group, nor was it investigated in Jataí (Souza et al. 2023) and Londrina (Torrico et al. 2020), making it impossible to make comparisons. However, it is worth noting that kittens under six months old are commonly more affected compared to adult animals (Bouzid et al. 2015).

In our study, using the Faust technique, *Giardia* spp. cysts were observed in the feces of 193 cats, and through the immunochromatographic assay, coproantigens of the protozoan were detected in 203 samples, demonstrating an almost perfect agreement between the diagnostic tests (Online Resource 1). These results corroborate previous studies, which detected an agreement between the parasitological examination and the immunochromatographic assay in the diagnosis of *Giardia* spp. in cats, dogs and humans (de Mendonça Uchôa et al. 2018). However, the immunochromatographic assay detected a higher occurrence (5% [7/140]) of the aforementioned protozoan when compared to the coproparasitological examination (1.4% [2/140]) in felines in Nigeria (Adeiza et al. 2024). However, in none of these studies was Cohen’s *Kappa* coefficient calculated, making it impossible for us to make further comparisons.

Finally, it is worth noting that, despite collecting only one sample for the diagnosis of *Giardia* spp., we identified a high prevalence of the aforementioned agent in felines in the municipality of Londrina, Paraná, Brazil. However, we cannot rule out the possibility of false negatives in our study, since the release of cysts along with the feces of the hosts can occur intermittently and even in low quantities, thus favoring underdiagnosis and an underestimated prevalence (Uchôa et al. 2017).

## Conclusion

In this research, we identified a high prevalence of *Giardia* spp. in felines, being significantly higher in adult and non-domiciled animals. Regarding diagnostic methods, the Faust technique showed almost perfect agreement with the immunochromatographic assay, thus increasing the accuracy in the diagnosis of this protozoan.

## Supplementary Information

Below is the link to the electronic supplementary material.

Supplementary Material 1

Supplementary Material 2

## Data Availability

No datasets were generated or analysed during the current study.
