# Supplementary material for: High prevalence of Giardia spp. in cats without diarrhea and agreement between diagnostic techniques in the municipality of Londrina, Paraná, Brazil
Source: Vet Res Commun. 2026 Jul 20;50(5):467. doi: 10.1007/s11259-026-11409-8 (PMC13384987; doi:10.1007/s11259-026-11409-8)
Supplement: Supplementary file 1 — Supplementary Material 1 [file 11259_2026_11409_MOESM1_ESM.docx]

**Online Resource 1 -** Comparison between the Faust technique and the immunochromatographic assay in the diagnosis of *Giardia* spp. in feline fecal samples from the municipality of Londrina, Paraná state, Brazil.

| **Diagnostic method** | | **Immunochromatographic Assay¹** | | **Agreement** | ***Kappa’*s value** |
| --- | --- | --- | --- | --- | --- |
|  |  |  |  |  |  |
|  |  | **Positive** | **Negative** |  |  |
| **Faust technique** | **Positive** | 193 | 0 | **96%** | ***0.907**** |
|  | **Negative** | 10 | 69 |  |  |

*Almost perfect agreement.

¹*Giardia* Ag VET FAST
