# Supplementary material for: High prevalence of Giardia spp. in cats without diarrhea and agreement between diagnostic techniques in the municipality of Londrina, Paraná, Brazil
Source: Vet Res Commun. 2026 Jul 20;50(5):467. doi: 10.1007/s11259-026-11409-8 (PMC13384987; doi:10.1007/s11259-026-11409-8)
Supplement: Supplementary file 2 — Supplementary Material 2 [file 11259_2026_11409_MOESM2_ESM.docx]

**Online Resource 2 –** Prevalence of Giardia spp. in relation to age group, rearing system and diagnostic techniques in felines from the municipality of Londrina, state of Paraná, Brazil.

| **Variables** | | **Nº of samples** | ***Giardia* Ag VET FAST (%)** | **Faust technique (%)** |
| --- | --- | --- | --- | --- |
| Domiciled | Kittens | 12 | 6 (50) | 0 (0) |
|  | Adults | 56 | 22 (39.3) | 21 (37.5) |
| **Total** | | 68 | 28 (41.2) | 21 (30.9) |
| NGOs | Kittens | 8 | 3 (37.5) | 0 (0) |
|  | Adults | 196 | 172 (87.8) | 172 (87.8) |
| **Total** | | 204 | 175 (85.8) | 172 (84.3) |

Nº - Number

NGOs - Non-Governmental Organizations
